# Data on induction of brown/beige adipocytes in mouse retro-orbital adipose tissues

**DOI:** 10.1016/j.dib.2019.104659

**Published:** 2019-10-15

**Authors:** Makoto Sugiyama, Masayuki Funaba, Osamu Hashimoto

**Affiliations:** a Faculty of Veterinary Medicine, Kitasato University, School of Veterinary Medicine, Towada, Aomori 034-8628, Japan; b Division of Applied Biosciences, Graduate School of Agriculture, Kyoto University, Kitashirakawa Oiwakecho, Kyoto 606-8502, Japan

**Keywords:** Eye, Metabolism, Ocular disorder, Retrobulbar, Thermogenesis

## Abstract

The data presented here are related to the research article entitled “Inducible brown/beige adipocytes in retro-orbital adipose tissues” [1]. Brown and beige adipocytes dissipate stored energy through the generation of heat by using mitochondrial uncoupling protein 1 (Ucp1), which is a mammalian brown/beige adipocyte-specific protein that promotes proton leakage across the inner mitochondrial membrane. Both cells up-regulate Ucp1 expression in response to β-adrenergic receptor activation such as cold exposure. Here, we provide data on induction of Ucp1 positive cells in retro-orbital white adipose tissues (WAT) from cold exposed both male and female mice. The fluctuation of eye surface temperature was monitored during the cold exposure. In addition, distribution of tyrosine hydroxylase positive bundles was observed in the retro-orbital WAT from the mice.

**Table:** Specifications Table

Subject area	Biology
More specific subject area	Adipocyte
Type of data	Image, graph
How data was acquired	Olympus BH2 microscope, FLIR i7 Infrared thermal imaging camera and NIH image J 1.48 Mac OS X
Data format	Raw
Experimental factors	Mice were kept at 4 °C for 48hr.
Experimental features	Temperature of the eye surfaces was measured and the orbital adipose tissues were obtained for histology.
Data source location	Kitasato University, School of Veterinary Medicine, Towada, Japan
Data accessibility	Data is within this article

**Table:** 

**Value of the Data** • M. Sugiyama, D. Shindo, N. Kanada, T. Ohzeki, K. Yoshioka, M. Funaba, O. Hashimoto, Inducible brown/beige adipocytes in retro-orbital adipose tissues. *Exp. Eye Res.* 184 (2019) 8–14 [1]. • The data will aid in better understanding of the function of brown/beige adipocytes in retro-orbital WAT. • The data indicated that retro-orbital WAT may worth being further examined in the future as they might present means of diagnostic and treatment options for metabolic disorders. • The data could be useful to provide potential therapy for ocular disorder such as cold cataract and accommodative dysfunction.

## 1 Data

**Fig. 1:**
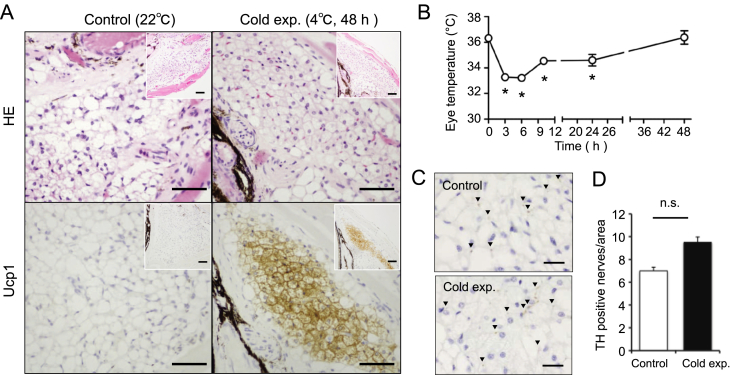
**The surface temperatures of eyes and the expression of Ucp1 in retro-orbital WAT in female mice**. (A) Histological analysis of retro-orbital white adipose tissue (WAT) from female mice (12 weeks old) exposed to 4 °C for 48 h. Upper panels are retro-orbital WAT sections with hematoxylin-eosin (HE) stain. Lower panels are immunohistochemistry of sections for Ucp1. Inserts indicate low magnification of the sections. Representative data are shown. Bar, 50 μm. (B) The surface temperatures of eyes were measured at the indicated points during cold exposure (upper panel). *n =* 4. Values are mean ± SEM. *P < 0.05 vs. data at 0 h. (C) Immunohistochemical stain of tyrosine hydroxylase (TH) in retro-orbital WAT. Positive reactions of TH were observed in retro-orbital WAT from female mice (12 weeks old). Upper panel, mice maintained at 22 °C. Lower panel, mice exposed to 4 °C for 48 h. Arrow heads indicate the positive reactions. Representative data are shown. Bar, 20 μm. (D) The number of TH positive nerves was analyzed by the NIH image. Values are mean ± SEM.

**Fig. 2:**
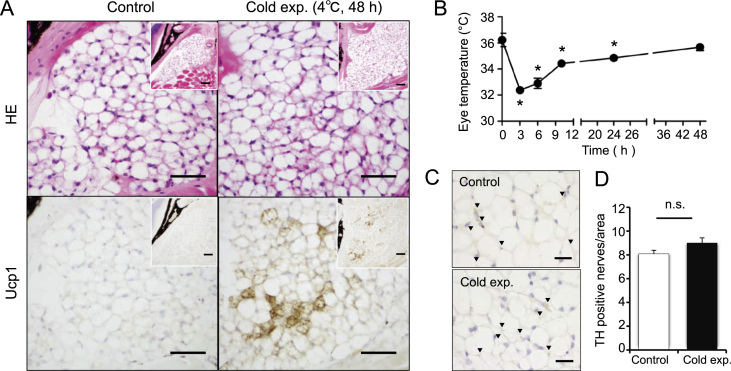
**Cold exposure induces Ucp1 adipocytes in retro-orbital WAT and enhances the surface temperature of eyes in 22 weeks old male mice**. (A) Histological analysis of retro-orbital white adipose tissue (WAT) from mice (22 weeks old) exposed to 4 °C for 48 h. Upper panels are retro-orbital WAT sections with hematoxylin-eosin (HE) stain. Lower panels are immunohistochemistry of sections for Ucp1. Inserts indicate low magnification of the sections. Representative data are shown. Bar, 50 μm. (B) The surface temperatures of eyes were measured at the indicated points during cold exposure (upper panel). *n =* 4. Values are mean ± SEM. *P < 0.05 vs. data at 0 h. (C) Immunohistochemical stain of tyrosine hydroxylase (TH) in retro-orbital WAT. Positive reactions of TH were observed in retro-orbital WAT male mice (22 weeks old). Left panel, mice maintained at 22 °C. right panel, mice exposed to 4 °C for 48 h. Arrow heads indicate the positive reactions. Representative data are shown. Bar, 20 μm. (D) The number of TH positive nerves was analyzed by the NIH image. Values are mean ± SEM.

Data shown in this article are related to the research article titled “Inducible brown/beige adipocytes in retro-orbital adipose tissues” [1]. Ucp1-positive adipocytes with multilocular lipid droplets emerged in retro-orbital WAT in cold exposed 12 weeks aged female (Fig. 1A) and 22 weeks aged male (Fig. 2A) mice. Furthermore, eye surface temperature remained within a physiological range during the cold challenge Fig. 1, Fig. 2B). The raw data related to Fig. 1, Fig. 2B were shown in Supplementary Data 1 and 2, respectively. Tyrosine hydroxylase positive reaction was observed in the retro-orbital WAT (Fig. 1, Fig. 2C). The number of tyrosine hydroxylase positive bundles in the cold exposed retro-orbital WAT was comparable with the control retro-orbital WAT (Fig. 1, Fig. 2D). The raw data related to Fig. 1, Fig. 2D were shown in Supplementary Data 3 and 4, respectively.

## 2 Experimental design, materials and methods

### 2.1 Animals

C57BL/6J mice (CLEA Japan Inc. Tokyo, Japan) were maintained in a 12-h light–dark cycle at 22 ± 4 °C, and given a normal diet (CE-2; CLEA Japan Inc.) and water *ad libitum*. Surface temperature of eyes was measured by using an infrared thermal imaging camera (Model: FLIR i7; Flir Systems, Inc., Wilsonville, OR, USA) at a distance of 30 cm between the eye and the camera. For cold exposure, mice were kept at 4 °C in a refrigerator with a temperature regulator (Sanyo Medical, Panasonic Corp., Tokyo, Japan). All experiments were performed between 13:00 and 16:30 during the light cycle. Experimental procedures and animal care were performed in accordance with the requirements of the Institutional Animal Care Committee at Kitasato University, in compliance with National Institutes of Health guidelines (approval no. 18-041).

### 2.2 Histological analysis

Mouse retro-orbital WATs were fixed in Bouin's fluid and embedded in paraffin. Four-micrometer sections were affixed to slides and stained with hematoxylin and eosin (HE). For immunohistochemistry, deparaffinized sections were incubated with H_2_O_2_, blocked with 10% normal goat serum, incubated with a rabbit polyclonal antibody to Ucp1 (3 μg/mL, No. ab10983; Abcam, Cambridge, UK) or tyrosine hydroxylase (1 μg/mL, No. AB152; Millipore, CA, USA) overnight at 4 °C, and then visualized with 3,3′-diaminobenzidine tetrahydrochloride by using the Histofine Simple Stain MAX-PO kit (Nichirei, Tokyo, Japan), as previously described [2]. For estimation of tyrosine hydroxylase-positive bundles in retro-orbital WATs, arbitrary fields of view observed under a 40× objective lens (approximately 4 × 10^4^ μm^2^/tissue) were analyzed by using the NIH image J 1.48 Mac OS X (National Institutes of Health, Maryland, USA) as described previously [2].
